# Supplementary material for: Identification of CCZ1 as an essential lysosomal trafficking regulator in Marburg and Ebola virus infections
Source: Nat Commun. 2023 Oct 25;14:6785. doi: 10.1038/s41467-023-42526-6 (PMC10600203; doi:10.1038/s41467-023-42526-6)
Supplement: Supplementary file 1 — Supplementary Information [file 41467_2023_42526_MOESM1_ESM.pdf]

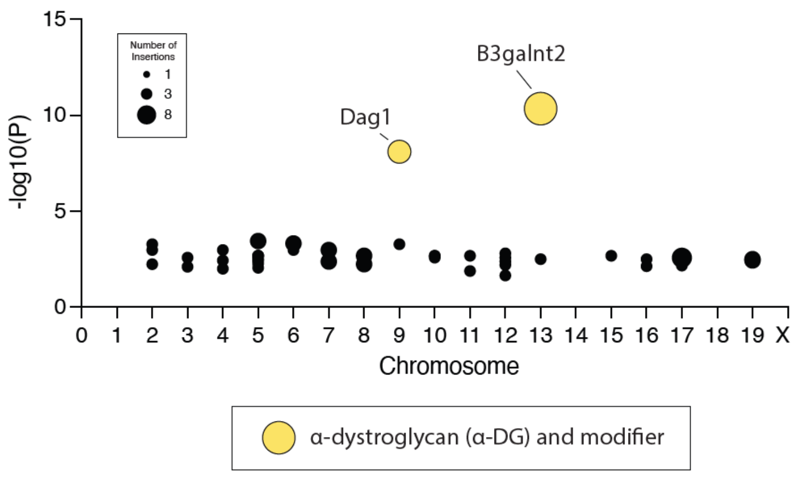

Supplementary figure 1. Identification of VSVΔG/LASVGPC genes of resistance.

Bubble-plot showing hits identified from the VSVΔG/LASVGPC gene-trap insertion screen. Hits are shown as individual dots, grouped by chromosomal location and stratified along the y-axis by statistical significance of gene insertions found in the screen over background. Dot size indicated the number of unique insertions found. Genes with more than four unique insertions are color-coded by functional groups and labeled. Significant genes were selected using a binomial test of disruptive insertions compared to the undisruptive insertions and was done for each gene as previously described[14,53,54,55].

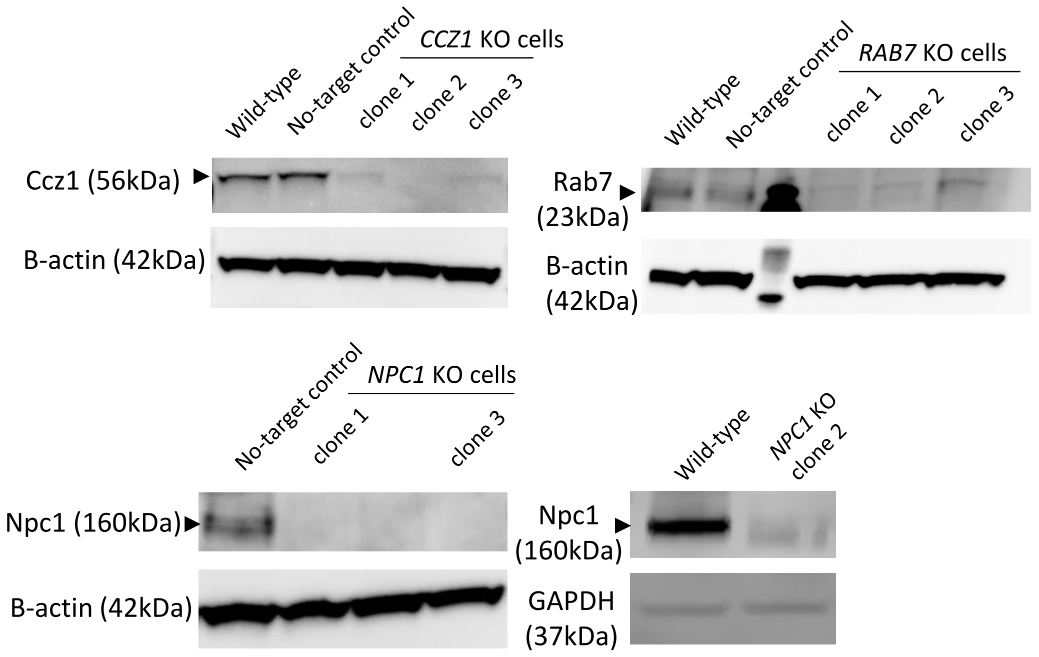

Supplementary figure 2. Knockout of *CCZ1*, *NPC1* and *RAB7* in A549 clones

Western-blot were runned on cell lysates to detect CCZ1, NPC1 or RAB7 proteins in knocked out A549 clones.

The experiment was repeated twice with similar results. Uncropped blots in Source Data.

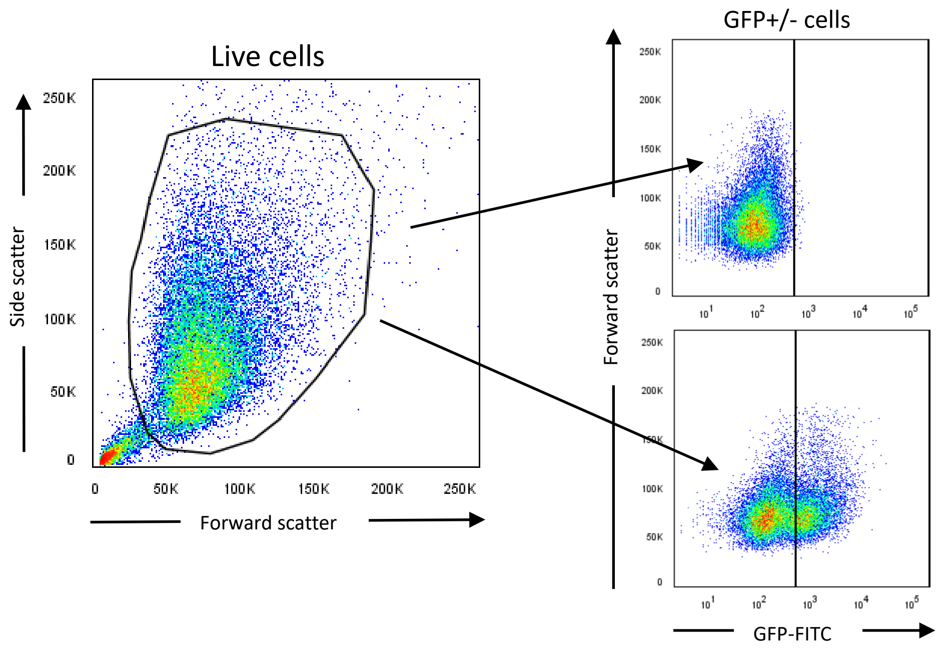

Supplementary Figure 3. Flow cytometry gating strategies.
Representative FACS plots show the gating strategy for GFP positive and negative cells. Debris and doublets (left) were excluded from the analysis.
The cells were then gated on GFP negative and positive cells (top right) based on a mixture of GFP negative cells (WT No Target, CCz1 KO, NPC1 KO and Rab7 KO). This gate was applied to all other samples in the experiment (bottom right). The same strategy was used at each time point.

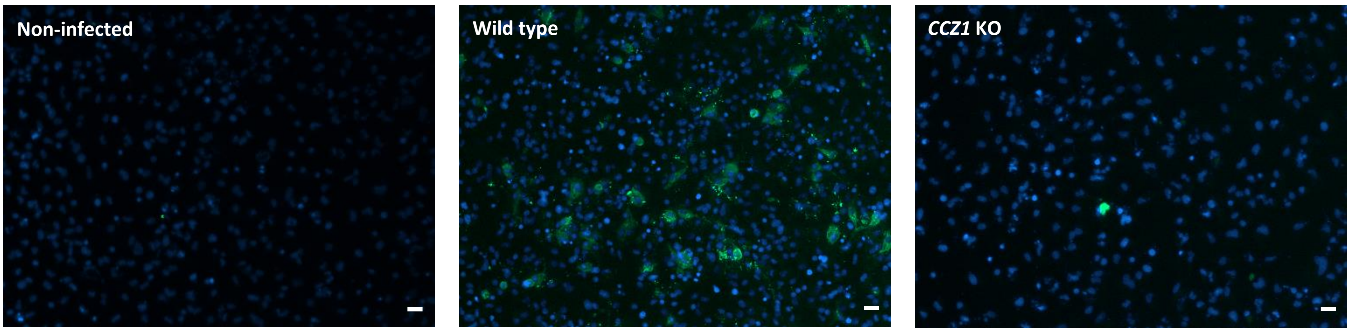

Supplementary figure 4. A549 cells infected with filovirus

Immunostaining analysis of filovirus infected cells. Scale: 10μM

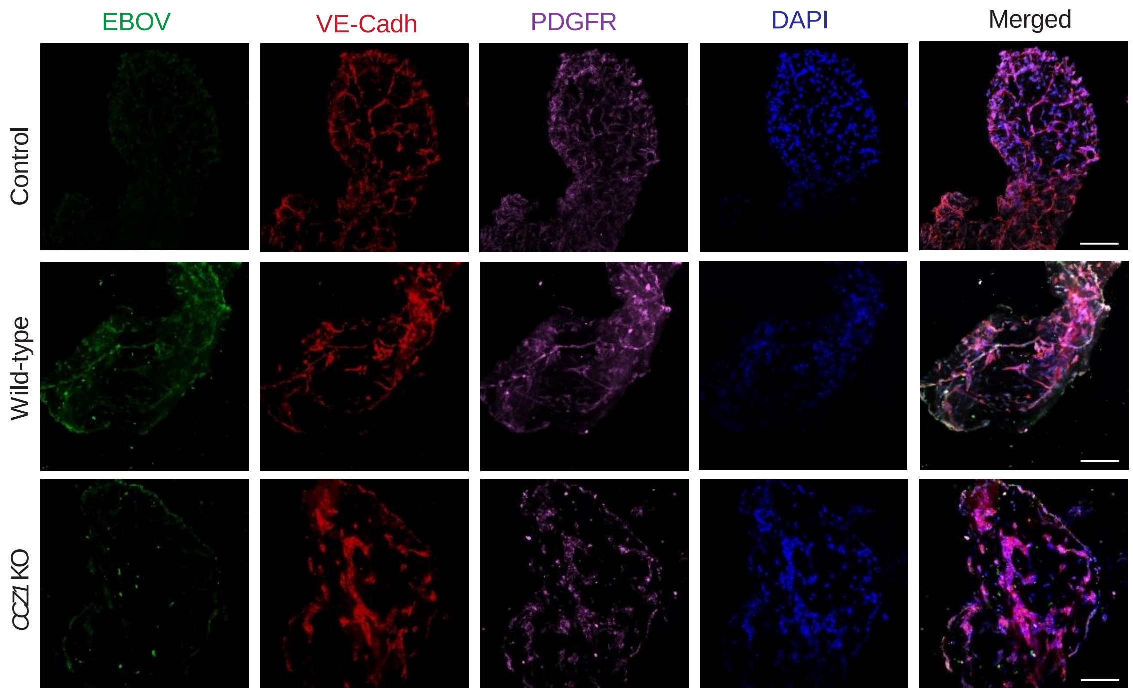

Supplementary Figure 5: Blood-vessels organoids KO for CCZ1 show less EBOV infection as highlighted by the qRT-PCR data (Figure 7d) Scale bar: 100µm. Blood-vessels organoids were stained for EBOV-GP (green), VE-Cadherin as a marker for endothelium (red), PDGFR as a marker for pericyte and DAPI (nuclei/blue).

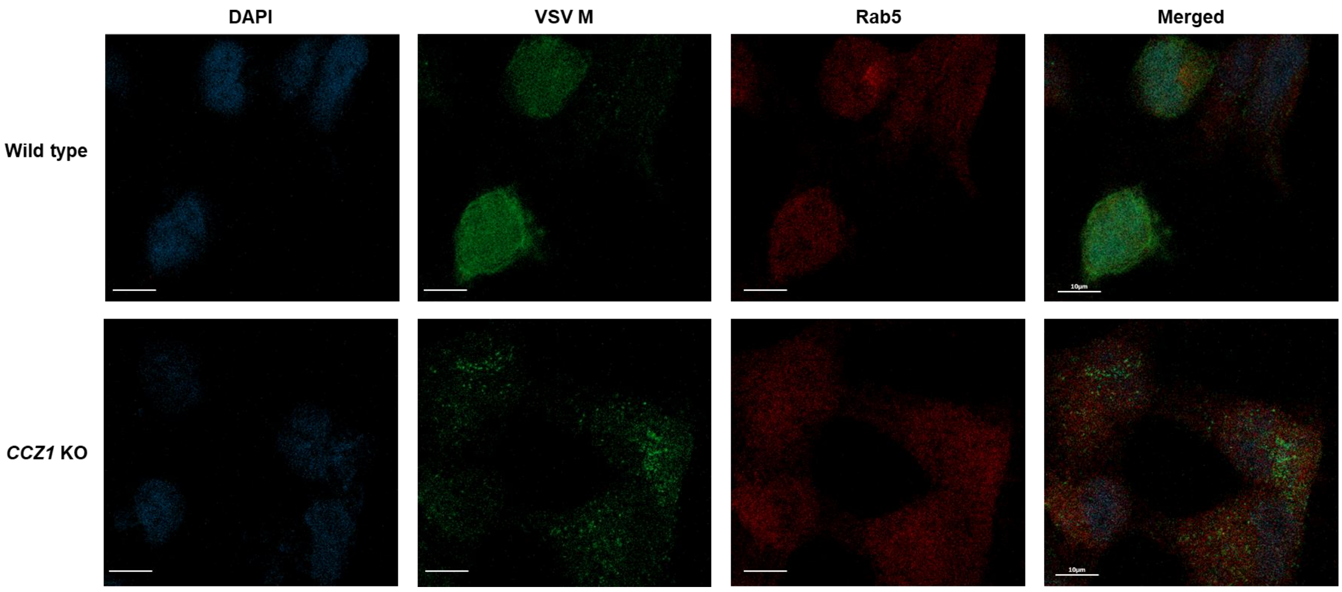

Supplementary Figure 6: Confocal microscopy analysis of wild type and *CCZ1* KO A549 cells infected with VSVΔG/MARVGP. Scale bar: 10µm. Cells were stained for VSVΔG/MARVGP (green), Rab5 as a marker of early endosome (red) and DAPI (nuclei/blue).

sampleID	chrom	pos	mut	reads	allele frequency	gene	transcript length	SNP annotation
COV2HYE2#15	chr5	144759133	C>A	48	100%	Ccz1	1443	Stop gained p.Gly283*
COV2HYE2#22	chr5	144771936	G>T	37	100%	Ccz1	1443	Stop gained p.Tyr132*
COV2HYE2#23	chr5	144752500	C>T	69	100%	Ccz1	1443	Splice acceptor variant
COV2HYE2#24	chr5	144773670	A>G	38	90%	Ccz1	1443	Splice donor variant
COV2HYE2#25	chr5	144773695	C>A	21	100%	Ccz1	1443	Stop gained p.Glu95*
COV2HYE2#26	chr5	144759426	G>A	52	100%	Ccz1	1443	Stop gained p.Gln275*
COV2HYE2#28	chr5	144764944	A>T	139	99%	Ccz1	1443	Stop gained p.Tyr220*
COV2HYE2#30	chr5	144762244	A>T	139	100%	Ccz1	1443	Splice donor variant
COV2HYE2#33	chr5	144773670	A>C	39	97%	Ccz1	1443	Splice donor variant

Supplementary figure 7. Haploid cells chemical mutagenesis screening

AN3-12 cells were chemically mutagenized and the obtained library was infected with SARS-CoV-2. This screening highlighted CCZ1 as a factor involved in SARS-CoV-2 infection.

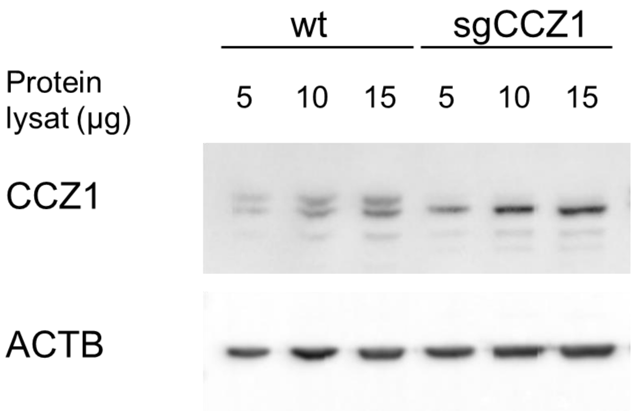

Supplementary figure 8. *CCZ1* knockout generation in Vero E6 cells.

Western Blot was used to probe for CCZ1 protein expression levels using Anti-CCZ1 antibody (HPA045114,Sigma-Aldrich). As loading control Anti-Beta-actin antibody was used (8H10D10, Cell Signaling Technology)
