# Supplementary material for: Identification of CCZ1 as an essential lysosomal trafficking regulator in Marburg and Ebola virus infections
Source: Nat Commun. 2023 Oct 25;14:6785. doi: 10.1038/s41467-023-42526-6 (PMC10600203; doi:10.1038/s41467-023-42526-6)
Supplement: Supplementary file 3 — Source data [file 41467_2023_42526_MOESM3_ESM.zip › Source data/Supplementary uncropped WB.pdf]

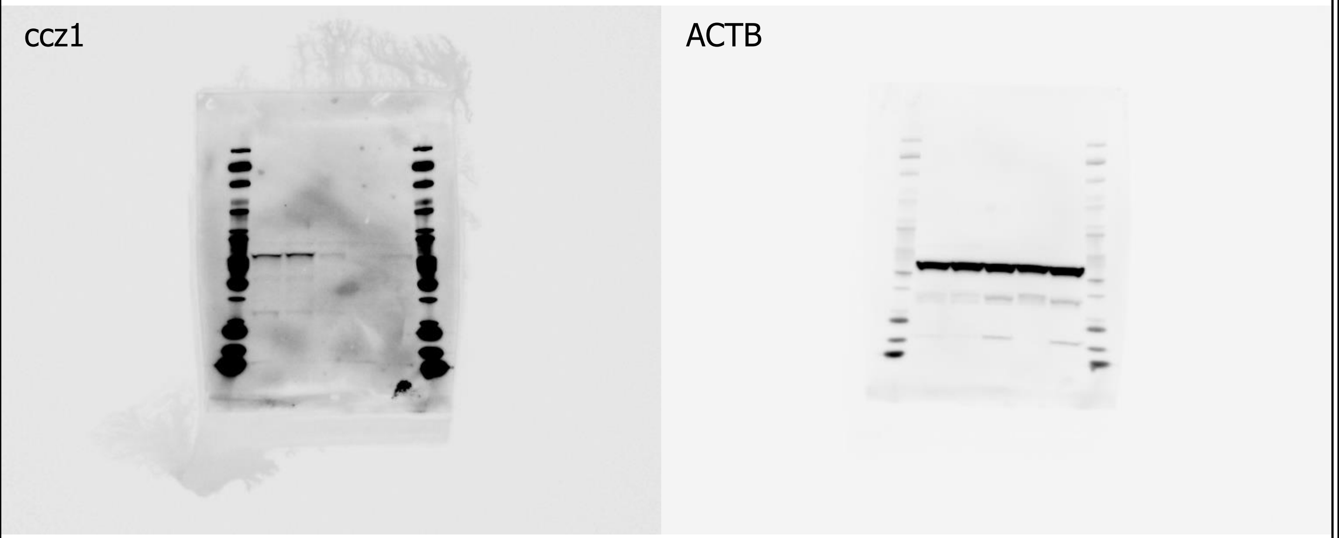

Haploid cells (suppl figure 2)
Staining:
CCZ1: Rabbit anti-Human CCZ1 antibody (1:500) (Sigma-Aldrich)
ACTB: Mouse anti-actin beta antibody (1:2000) (Thermofisher)
Ladder: PageRuler unstained protein ladder (Thermofisher)

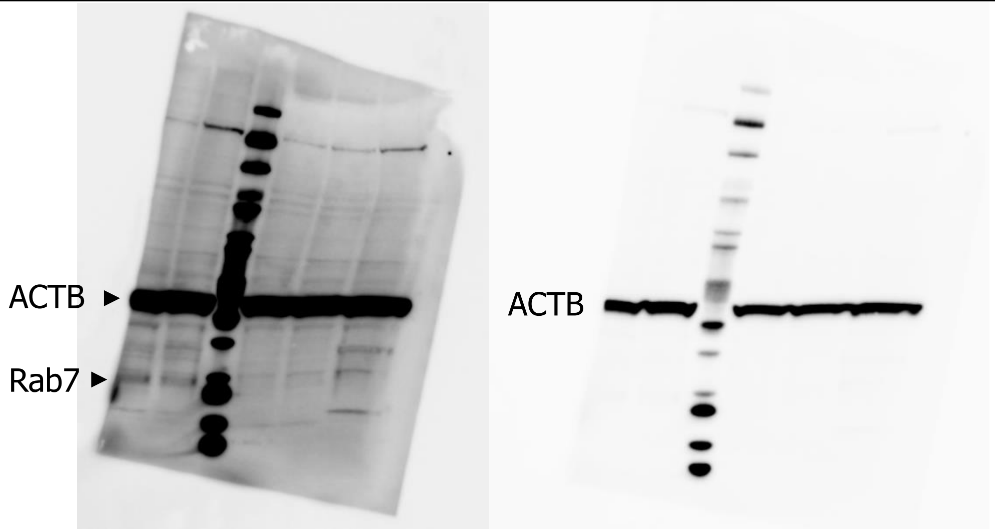

Haploid cells (suppl figure 2)
Staining:
Rab7: Mouse monoclonal anti-human Rab7 antibody (1:2000) (Sigma-Aldrich)
ACTB: Mouse anti-actin beta antibody (1:2000) (Thermofisher)
Ladder: PageRuler unstained protein ladder (Thermofisher)

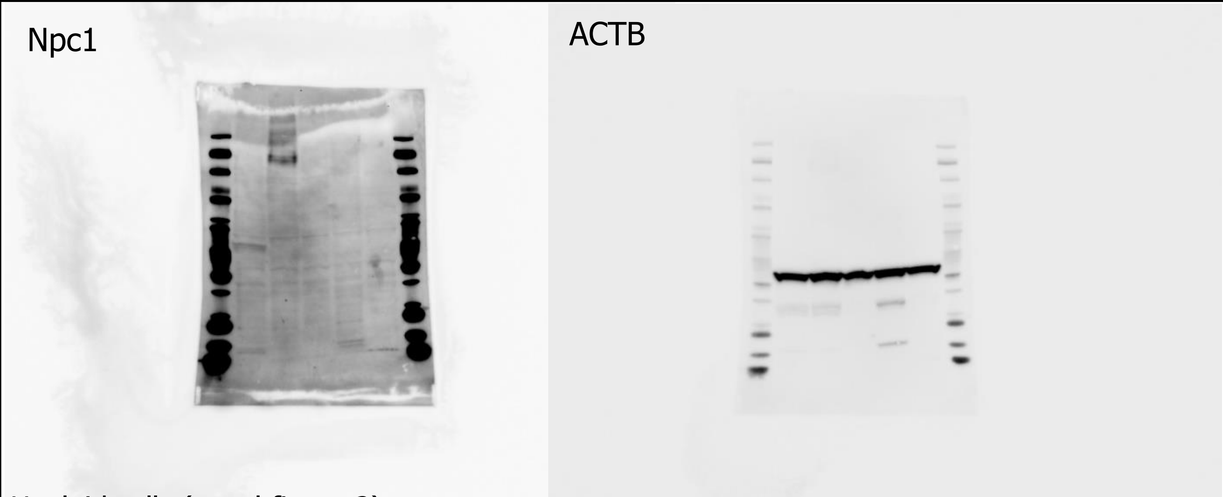

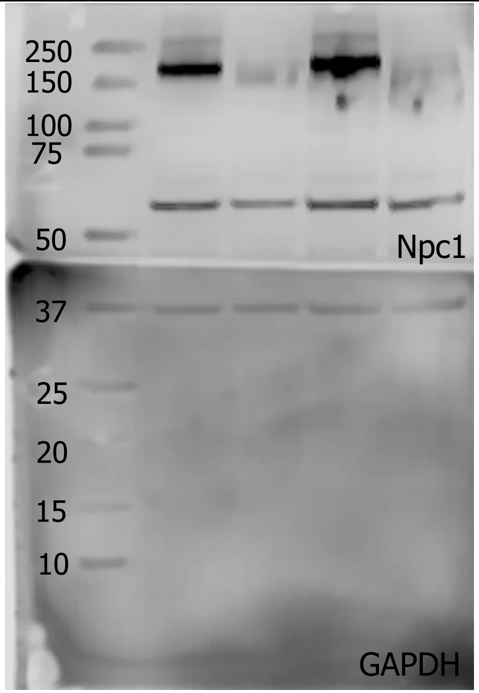

Haploid cells (suppl figure 2)
Staining:
Npc1: Rabbit anti-Human NPC1 antibody (1:1000) (Sigma-Aldrich)/ Novus NB400-148 (1/1000)
ACTB: Mouse anti-actin beta antibody (1:2000) (Thermofisher)
GAPDH: mouse anti-GAPDH antibody (1/5000) (Abcam)
Ladder: PageRuler unstained protein ladder (Thermofisher)
